# Creation and Magnetic Study of Ferrites with Magnetoplumbite Structure Multisubstituted by Al^3+^, Cr^3+^, Ga^3+^, and In^3+^ Cations

**DOI:** 10.3390/nano12081306

**Published:** 2022-04-11

**Authors:** Vladimir E. Zhivulin, Daria P. Sherstyuk, Olga V. Zaitseva, Natalia A. Cherkasova, Denis A. Vinnik, Sergey V. Taskaev, Evgeny A. Trofimov, Sergei V. Trukhanov, Siarhei I. Latushka, Daria I. Tishkevich, Tatiana I. Zubar, Alex V. Trukhanov

**Affiliations:** 1 Laboratory of Single Crystal Growth, South Ural State University, 76, Lenin Av., 454080 Chelyabinsk, Russia; zhivulinve@mail.ru (V.E.Z.); sherstiukd@susu.ru (D.P.S.); zaitcevaov@susu.ru (O.V.Z.); Cherkasova.N.A@yandex.ru (N.A.C.); denisvinnik@gmail.com (D.A.V.); tea7510@gmail.com (E.A.T.); div_tishkevich@mail.ru (D.I.T.); ti_zubar@mail.ru (T.I.Z.); truhanov86@mail.ru (A.V.T.); 2 Faculty of Physics, Chelyabinsk State University, 129, Bratiev Kashirinykh St., 454001 Chelyabinsk, Russia; sv_taskaev@mail.ru; 3 Laboratory of Magnetic Films Physics, SSPA “Scientific and Practical Materials Research Centre of NAS of Belarus”, 19, P. Brovki Str., 220072 Minsk, Belarus; si_latushka@mail.ru; 4 Department of Electronic Materials Technology, National University of Science and Technology MISiS, 119049 Moscow, Russia

**Keywords:** M-type hexaferrites, structure, high-entropy oxides, magnetization, anisotropy

## Abstract

Multisubstituted barium ferrites with a magnetoplumbite structure were obtained by the method of solid-phase reactions with ferritization and pre-firing. Three-charged, mainly diamagnetic cations Al^3+^, Cr^3+^, Ga^3+^, and In^3+^ were chosen as substituents for the Fe^3+^ iron cations, the proportion of which in solid solutions did not exceed 50%. The values of the configurational mixing entropy were calculated for all the compositions. A slight deviation of the chemical composition of the obtained solid solutions from the nominal value was established by the energy-dispersive X-ray spectroscopy method. The phase purity and values of the unit cell parameters were refined from X-ray scattering data using full-profile analysis in the Rietveld method. A non-monotonic behavior of the unit cell parameters as a function of the B-sub-lattice average ionic radius of the magnetoplumbite structure was found. A minimum unit cell volume of ~667.15 Å^3^ was found for the composition BaFe_6.11_Al_1.56_Cr_2.17_Ga_2.16_O_19_ with a B-sub-lattice average ionic radius of ~7.449 Å. The average crystallite size varied within 5.5–6.5 μm. The temperature and field dependencies of the magnetization have been measured. The values of the saturation magnetization, residual magnetization, hysteresis loop squareness, and coercivity at 50 K and 300 K were extracted from the experimental data. Using the Law of Approach to Saturation, the magnetic crystallographic anisotropy coefficient and anisotropy field were calculated. Multisubstitution leads to a significant decrease in such magnetic parameters as the magnetic ordering temperature and spontaneous magnetization at both temperatures. The maximum magnetic ordering temperature of ~297.7 K was found for the composition BaFe_5.84_Ga_6.19_O_19_ with a B-sub-lattice average ionic radius of ~7.586 Å in a field of 500 Oe. A maximum saturation magnetization of ~24.7 emu/g was found for the composition BaFe_5.84_Ga_6.19_O_19_ with a B-sub-lattice average ionic radius of ~7.586 Å at 50 K. A maximum hysteresis loop squareness of ~0.72 was found for the composition BaFe_6.11_Al_1.56_Cr_2.17_Ga_2.16_O_19_ with an average ionic radius of ~7.449 Å at 50 K. A maximum magnetic crystallographic anisotropy coefficient of ~2.09 × 10^5^ Erg/g was found for the composition BaFe_6.19_Al_1.25_Cr_1.57_Ga_1.74_In_1.26_O_19_ with a B-sub-lattice average ionic radius of ~7.706 Å at 50 K. The frustrated magnetic state including the nano-sized clusters with an average diameter in the range of 50–200 nm was established from the results of measuring the ZFC and FC temperature magnetizations. The interpretation of the obtained experimental data is carried out taking into account the increased stability of high-entropy phases and regular changes in the intensity of the Fe^3+^(Al^3+^, Cr^3+^, Ga^3+^, In^3+^)–O^2−^–Fe^3+^(Al^3+^, Cr^3+^, Ga^3+^, In^3+^) indirect superexchange interactions as a result of magnetic dilution of the iron sub-lattice in the magnetoplumbite structure.

## 1. Introduction

M-type hexaferrites with a magnetoplumbite structure were the subject of intense research almost a century ago [1] and, due to their properties—chemical inertness, mechanical strength, high values of the Curie temperature, coercive force, and anisotropy field—are widely used in various fields of technology [2,3,4]. In particular, these materials are used in magnetic circuits [5], in a number of high-frequency electronics components [6,7,8], in multiple-state memory elements [9], new functional sensors [10,11,12], etc.

The growing demands of consumers for the quantitative characteristics of the properties of compounds with the magnetoplumbite structure led to the idea of obtaining crystalline structures in which some of the iron cations would be substituted by other cations [13]. It has been repeatedly shown that such a substitution leads to a change in the structural characteristics and especially properties of the resulting solid solutions, in particular, to a change in the degree of uniaxial magnetic anisotropy, the magnitude of the coercive force, and the Curie temperature [14,15,16]. This opens up possibilities for controlling the properties of M-type hexaferrites by varying the degree of substitution and substituting cations. An impressive number of works have been devoted to the study of the effect of substitution of Fe^3+^ iron cations in the initial matrix of the M-type hexagonal ferrites on other three-charged cations. Of great interest are works on substitution with gallium [17,18,19], aluminum [20,21,22], chromium [23,24,25], and indium [26,27,28].

However, this approach has some disadvantages, primarily due to the fact that the solubility of individual doping cations in the magnetoplumbite crystal structure, as well as their ability to substitute iron cations, are limited. Our team has recently developed a modern method [29] that allows expanding the range of solid solutions of M-type hexaferrites with multicomponent substitution. The high configurational mixing entropy of iron cation sub-lattice should increase the stability of the crystal structure with a high degree of substitution. High values of the configuration entropy should contribute to an increase in the entropy of the phase as a whole, which will lead to a decrease in its Gibbs energy, and thus make the process of formation and existence of this phase more thermodynamically probable than the formation of other phases from the same elements [30].

In general, an approach related to the creation and study of high-entropy phases has emerged in the last two decades [31], primarily in relation to metal alloys, but in recent years this approach has been actively and effectively used to create new oxide phases [32]. The high-entropy oxide solid solutions with the structure of spinel [33], perovskite [34], garnet [35], etc. have already been obtained.

In a number of our works [36,37,38,39], for the first time in the world, high-entropy oxides with the magnetoplumbite structure were obtained and the electrical and magnetic properties of a number of representatives of this new group of solid solutions were studied. It was found that there is a correlation between the entropy state of the obtained solid solutions and the features of their crystal structure. It was shown that the magnetic properties depended critically on the entropy state and the intensity of indirect superexchange interactions. Magnetic phase separation was detected, which manifested itself in the coexistence of frustrated magnetic clusters in a matrix with a ferrimagnetic ordering. A strong relationship has also been demonstrated between the entropy state, magnetic, and electrical properties.

Interest in such compositions is due to the fact that there are areas of application for which it is desirable to obtain ceramics with the magnetoplumbite structure and an up to 50% degree of substitution of iron cations by diamagnetic ones. Obtaining such structures with one substituting element is not feasible for all the possible dopants. However, an increase in their number accompanied by a formal increase in the configuration mixing entropy of the sub-lattice formed by iron cations should contribute to the stabilization of the obtained structures [40]. At the same time, the question of how an increase in the amount of dopants while maintaining the degree of iron substitution affects the magnetic characteristics of the formed phase remains open. The study of the effects associated with an increase in the mixing entropy in such structures is of interest both from the theory point of view and from the point of view of applied developments in the field of creating new magnetic compositions.

The purpose of this work is to study the structure and magnetic characteristics of a series of barium hexaferrite samples with the constant 50% iron content and with a gradual increase in the number of substituting cations such as Al^3+^, Cr^3+^, Ga^3+^, and In^3+^.

## 2. Materials and Methods

### 2.1. Sample Preparation Methods

The studied samples were obtained by solid-phase synthesis. The oxides of iron Fe_2_O_3_, gallium Ga_2_O_3_, aluminum Al_2_O_3_, chromium Cr_2_O_3_, indium In_2_O_3_, and barium carbonate BaCO_3_ were used as initial components for the synthesis of samples. All the components used had a qualification not lower than the purity for analysis—analytical grade. The initial components were weighed in a given stoichiometric ratio and ground with an agate mortar. Table 1 shows the chemical formula of the composition and the mass content of the original oxides. In the last column of the same table, the results of the calculation of the configuration mixing entropy in the iron sub-lattice are computed using the formula:

(1) Smix=−∑xlnx,

where *x* is the fraction of cations of different elements in the sub-lattice.

The mixtures of oxides and carbonate obtained as a result of grinding were pressed into pellets with a diameter of 8 mm and a height of 5 mm using a hydraulic laboratory press in a steel mold. The pressing force was 5 tons. The resulting pellets were placed on a platinum substrate and sintered at a temperature of 1400 °C for 5 h [41]. The chemical reactions that occur during ferritization can be represented as:

BaCO_3_ + 3Al_2_O_3_ + 3Fe_2_O_3_ → BaFe_6_Al_6_O_19_ + CO_2_↑ (2)

BaCO_3_ + 3Cr_2_O_3_ + 3Fe_2_O_3_ → BaFe_6_Cr_6_O_19_ + CO_2_↑ (3)

BaCO_3_ + 3Ga_2_O_3_ + 3Fe_2_O_3_ → BaFe_6_Ga_6_O_19_ + CO_2_↑ (4)

BaCO_3_ + 3/2 Cr_2_O_3_ + 3/2 Al_2_O_3_ + 3Fe_2_O_3_ → BaFe_6_Al_3_Cr_3_O_19_ + CO_2_↑ (5)

BaCO_3_ + Cr_2_O_3_ + Al_2_O_3_ + Ga_2_O_3_ + 3Fe_2_O_3_ → BaFe_6_Al_2_Cr_2_Ga_2_O_19_ + CO_2_↑ (6)

BaCO_3_ + 3/4 Cr_2_O_3_ + 3/4 Al_2_O_3_ + 3/4 In_2_O_3_ + 3/4 Ga_2_O_3_ + 3Fe_2_O_3_ → → BaFe_6_Al_1.5_Cr_1.5_Ga_1.5_In_1.5_O_19_ + CO_2_↑. (7)

Conventionally, the chemical reaction can be divided into two stages. At the first stage, barium carbonate BaCO_3_ decomposes to barium oxide BaO with the release of carbon dioxide CO_2_. At the second stage, a reaction occurs between the initial oxides and the formation of complex barium hexaferrite.

### 2.2. Structure and Elemental Composition Study Methods

The samples obtained as a result of the chemical reaction were studied by X-ray phase analysis, electron microscopy, and elemental microanalysis. The X-ray diffraction (XRD) patterns in the angular range of 5°–90° were recorded with a scanning rate of 1°/min using a Rigaku model Ultima IV powder diffractometer using Cu-K_α_ radiation filtered by a Ni filter. The phase content was checked and the unit cell parameters at room temperature were calculated. The calculation of the unit cell parameters was carried out by the method of full-profile Rietveld analysis [42] in the Match 3.12 software package. The chemical composition was explored by the EDX (Oxford INCA X-max 80). The SEM measurements were realized for the microstructure images. The SEM photos were treated and the average particle size was computed for each sample [43].

### 2.3. Magnetic Study Methods

The magnetization measurements have been realized using a SQUID magnetometer [44]. The field investigations of a magnetization were implemented from 3 T down to −3 T and back at two temperatures of 50 K and 300 K. For revelation of the spontaneous magnetization, the Law of Approach to Saturation (LAS) [45] was employed. The residual magnetization, loop squareness, and coercivity were extracted. The computation of the magnetic crystalline anisotropy coefficient and anisotropy field was performed. The temperature investigations of a magnetization were implemented from 50 K up to 300 K in fields of 0.01 T and 0.05 T. The minimum point of the magnetization derivative was adopted as the magnetic ordering temperature [46].

## 3. Results and Discussion

### 3.1. Study of the Crystal Structure

**Figure 1:**
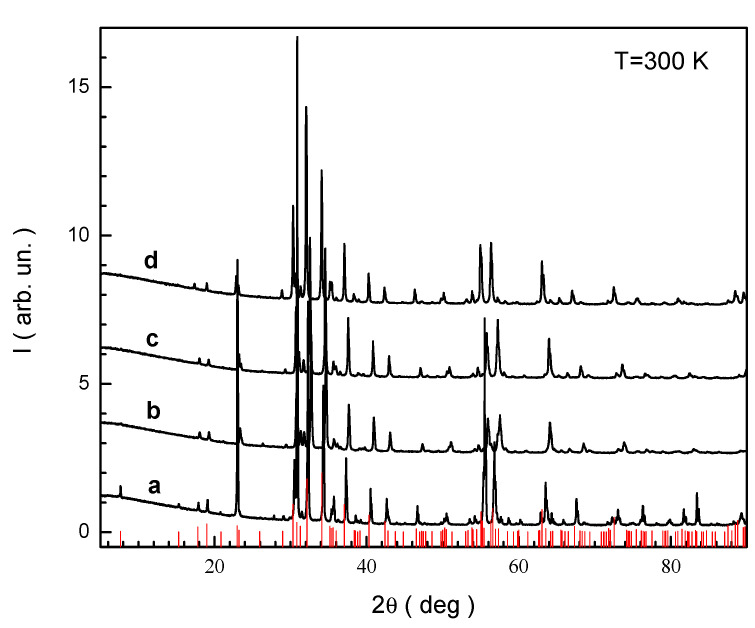
X-ray diffraction patterns at 300 K for the (a) BaFe_5.84_Ga_6.19_O_19_, (b) BaFe_6.26_Al_2.27_Cr_3.47_O_19_, (c) BaFe_6.11_Al_1.56_Cr_2.17_Ga_2.16_O_19_, (d) BaFe_6.19_Al_1.25_Cr_1.57_Ga_1.74_In_1.26_O_19_. Red bars are the Braggs reflections for the initial BaFe_12_O_19_ compound (PDF#43-0002).

The phase composition and unit cell parameters of all the obtained samples were determined by X-ray diffraction. The data obtained are shown in Figure 1. Samples with a given composition of BaFe_6_Al_6_O_19_ and BaFe_6_Cr_6_O_19_, according to X-ray diffraction and elemental microanalysis, consist of more than one phase. Along the phase of ~96wt% with the magnetoplumbite structure, there are phases with other structures. Therefore, it is inappropriate to study the magnetic characteristics of these samples.

Figure 1 shows XRD patterns of single-phase samples. The red bars indicate the literature data [3] for the BaFe_12_O_19_ barium hexaferrite. It can be seen from Figure 1 that all the reflections that appear in the X-ray diffraction patterns of the samples under study correspond to the ones given in the literature for the BaFe_12_O_19_ barium hexaferrite. It is also seen that the position of the reflections in the X-ray patterns of the samples under study is mainly shifted to the larger angle side, which indicates a decrease in inter-planar distances. This fact indicates changes in the parameters of the crystal lattice as a result of the substitution of iron cations by other cations that have different ionic radii. The unit cell parameters were calculated from the data of powder XRD patterns. Table 2 presents the calculation of the unit cell parameters of the obtained samples.

Table 2 shows that the unit cell parameters of the obtained samples differ from the parameters for the barium hexaferrite. The *a* parameter for all the samples has a lower value than for the unsubstituted hexaferrite. The *c* parameter for all the samples except for the BaFe_6.19_Al_1.25_Cr_1.57_Ga_1.74_In_1.26_O_19_ (d) composition has a smaller value.

The ratios of the parameters presented in Table 2 are in good agreement with the ratios of the radii of trivalent cations in the same coordination [47]:

r(Al^3+^,IV) = 0.535 Å < r(Cr^3+^,IV) = 0.615 Å < r(Ga^3+^,IV) = 0.620 Å < r(Fe^3+^,IV,HS) = 0.645 Å < < r(In^3+^,V) = 0.800 Å. (8)

Knowing the r(B^3+^) ionic radii of all the B^3+^ three-charged cations involved in the formation of the solid solutions and their concentration, it is possible to calculate the 〈r_B_〉 average ionic radius of the generalized B-sub-lattice of the magnetoplumbite structure formed by the iron cations. Such an average ionic radius of the B-sub-lattice is an integral quantitative characteristic of the intensity of the Fe^3+^(Al^3+^, Cr^3+^, Ga^3+^, In^3+^)–O^2−^–Fe^3+^(Al^3+^, Cr^3+^, Ga^3+^, In^3+^) indirect superexchange interactions. The smaller this radius, the closer the centers of exchange interaction and the higher the intensity of the exchange, and hence the stronger the ferrimagnetic properties.

**Figure 2:**
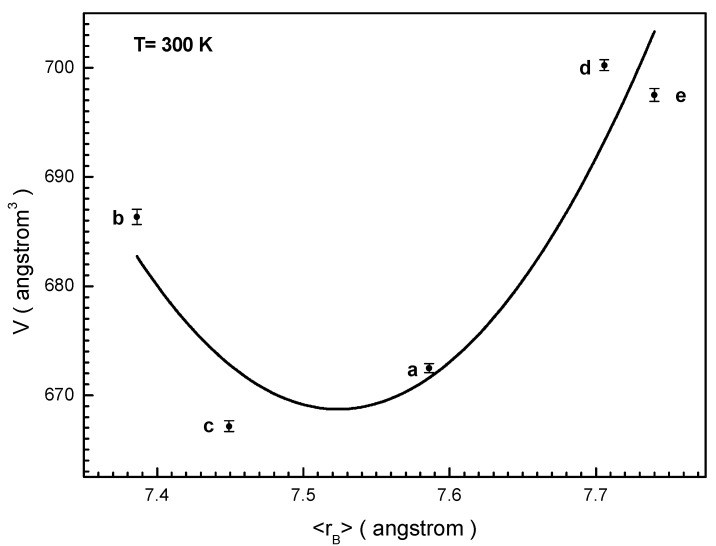
Dependence unit cell volume vs. 〈r_B_〉 average radius of B sub-lattice at 300 K for the (a) BaFe_5.84_Ga_6.19_O_19_, (b) BaFe_6.26_Al_2.27_Cr_3.47_O_19_, (c) BaFe_6.11_Al_1.56_Gr_2.17_Ga_2.16_O_19_, (d) BaFe_6.19_Al_1.25_Cr_1.57_Ga_1.74_In_1.26_O_19_, (e) initial BaFe_12_O_19_ compound. The line is the second order polynomial interpolation of experimental data.

Figure 2 shows the dependence of the *V* unit cell volume of the obtained solid solutions on the 〈r_B_〉 average ionic radius of the generalized B-sub-lattice. It is clearly seen that this dependence is not monotonic and has a minimum for the BaFe_6.26_Al_2.27_Cr_3.47_O_19_ (c) composition. The *V* unit cell volume for almost all the compositions is less than one for the initial BaFe_12_O_19_ barium hexaferrite. Only for the BaFe_6.19_Al_1.25_Cr_1.57_Ga_1.74_In_1.26_O_19_ (d) composition is the *V* unit cell volume slightly higher than that for the starting compound. This is explained by the influence of the largest ionic radius of the indium cation.

The non-monotonic dependence of the *V* unit cell volume with an increase in the average ionic radius of the generalized B-sub-lattice is explained by a change in the mechanism of filling substituent cations of different non-equivalent crystallographic positions. As is known, the structure of the barium hexaferrite contains singly degenerate octahedral position 2a and pentahedral position 2b, doubly degenerate tetrahedral position 4f_4_ and octahedral position 4f_6_, as well as six-fold degenerate position 12k [48]. Many data indicate that diamagnetic three-charged cations substitute iron cations predominantly in octahedral positions 2a and 12k [49,50,51]. However, depending on the substitution concentration, the tetrahedral and pentahedral sites can also be filled with such cations [52]. This filling mechanism leads to a significant decrease in the ionic radius, and a minimum can be observed in the presented dependence. Undoubtedly, a detailed study of the mechanism of the filling of iron positions by various cations has yet to be performed, however, such information on the dependence of the *V* unit cell volume on the 〈r_B_〉 average ionic radius of the generalized B-sub-lattice will be very useful below for interpreting the magnetic properties of the obtained solid solutions.

### 3.2. Study of the Surface Morphology and Elemental Composition

The elemental composition of the obtained samples was determined using an X-ray fluorescence analysis. Table 3 presents the results of elemental composition measurements and the gross formula calculated on their basis. From Table 3, it can be seen that the elemental composition of the obtained samples is in good agreement with the initially specified composition. According to our assumptions, the discrepancy between the initially specified composition and that calculated from EDX spectroscopy data is due to the error of the method.

**Figure 3:**
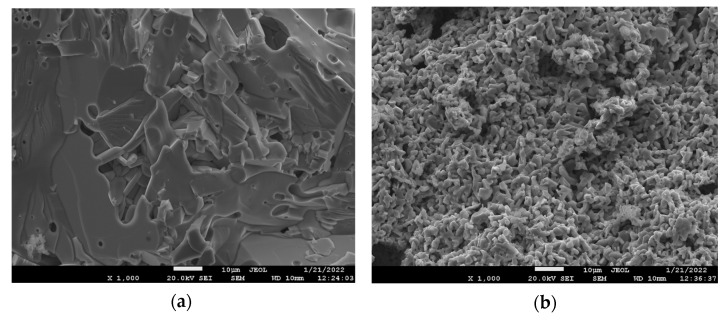

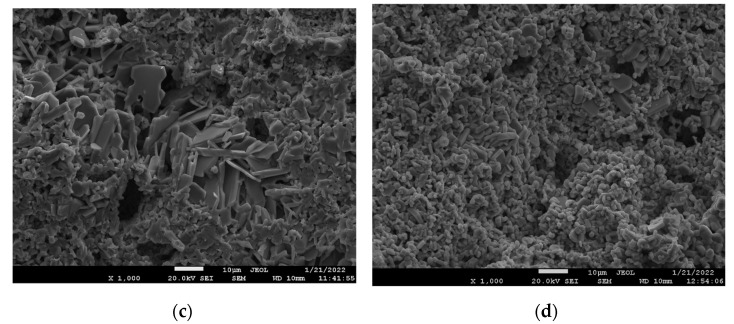
Scanning electron images of ceramic fracture for composition samples (**a**) BaFe_5.84_Ga_6.16_O_19_, (**b**) BaFe_6.26_Al_2.27_Cr_3.47_O_19_, (**c**) BaFe_6.11_Al_1.56_Cr_2.17_Ga_2.16_O_19_, (**d**) BaFe_6.19_Al_1.25_Cr_1.57_Ga_1.74_In_1.26_O_19_.

The surface morphology of the resulting ceramics was studied by scanning electron microscopy. Figure 3 shows images of ceramic fracture of the obtained samples. It can be seen from the figure that the samples have an inhomogeneous structure characteristic of ceramics. In the presented images, the particles are clearly visible, having a natural cut. The particles have the shape of a flattened hexagon, characteristic of the hexagonal ferrites.

These photographs show the particle size distribution in the range from 3 to 7 µm. Particles with sizes below and above these values were not observed at all. The relative amount of microparticles with a maximum size of 5 µm was ~45%. The average micro-particle size was in the range of 5.5–6.5 μm. The porosity of ceramics did not exceed ~3%.

### 3.3. Magnetic Measurements

**Figure 4:**
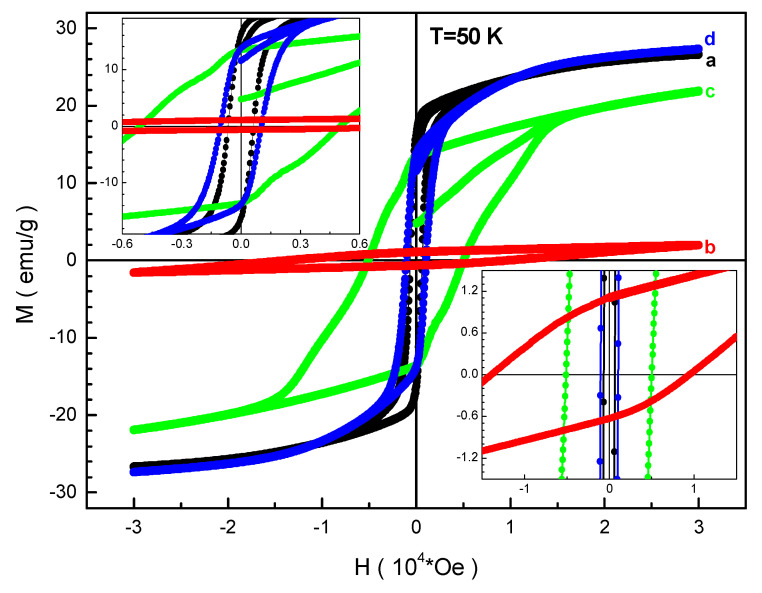
Field dependencies of magnetization at 50 K for the compositions (a) BaFe_5.84_Ga_6.16_O_19_, (b) BaFe_6.26_Al_2.27_Cr_3.47_O_19_, (c) BaFe_6.11_Al_1.56_Cr_2.17_Ga_2.16_O_19_, (d) BaFe_6.19_Al_1.25_Cr_1.57_Ga_1.74_In_1.26_O_19_.

**Figure 5:**
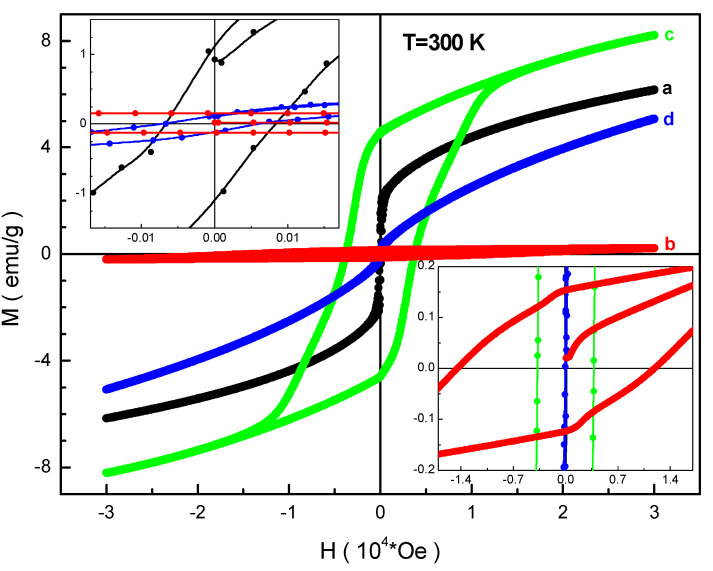
Field dependencies of magnetization at 300 K for the compositions (a) BaFe_5.84_Ga_6.16_O_19_, (b) BaFe_6.26_Al_2.27_Cr_3.47_O_19_, (c) BaFe_6.11_Al_1.56_Cr_2.17_Ga_2.16_O_19_, (d) BaFe_6.19_Al_1.25_Cr_1.57_Ga_1.74_In_1.26_O_19_.

The field dependencies of the magnetization of the obtained samples are shown in Figure 4 and Figure 5. It can be seen that these dependencies are closed loops due to a magnetic hysteresis. It should be noted that all the curves do not reach saturation in high fields up to 30 kOe.

At low temperatures, the BaFe_6.19_Al_1.25_Cr_1.57_Ga_1.74_In_1.26_O_19_ (d) composition has the maximum magnetization in the maximum field. The minimum magnetization at this temperature was recorded for the BaFe_6.26_Al_2.27_Cr_3.47_O_19_ (b) composition. The maximum coercivity also exists for the BaFe_6.26_Al_2.27_Cr_3.47_O_19_ (b) composition. At high temperature, the maximum magnetization in the maximum field was found for the BaFe_6.11_Al_1.56_Cr_2.17_Ga_2.16_O_19_ (c) composition. The minimum magnetization at high temperature was found for the BaFe_6.26_Al_2.27_Cr_3.47_O_19_ (b) composition. The maximum coercivity is also fixed for the BaFe_6.26_Al_2.27_Cr_3.47_O_19_ (b) composition. After analyzing these two figures, it is possible to extract all the necessary magnetic parameters and generalize them depending on the B-sub-lattice average ionic radius of the magnetoplumbite structure which is presented below.

**Figure 6:**
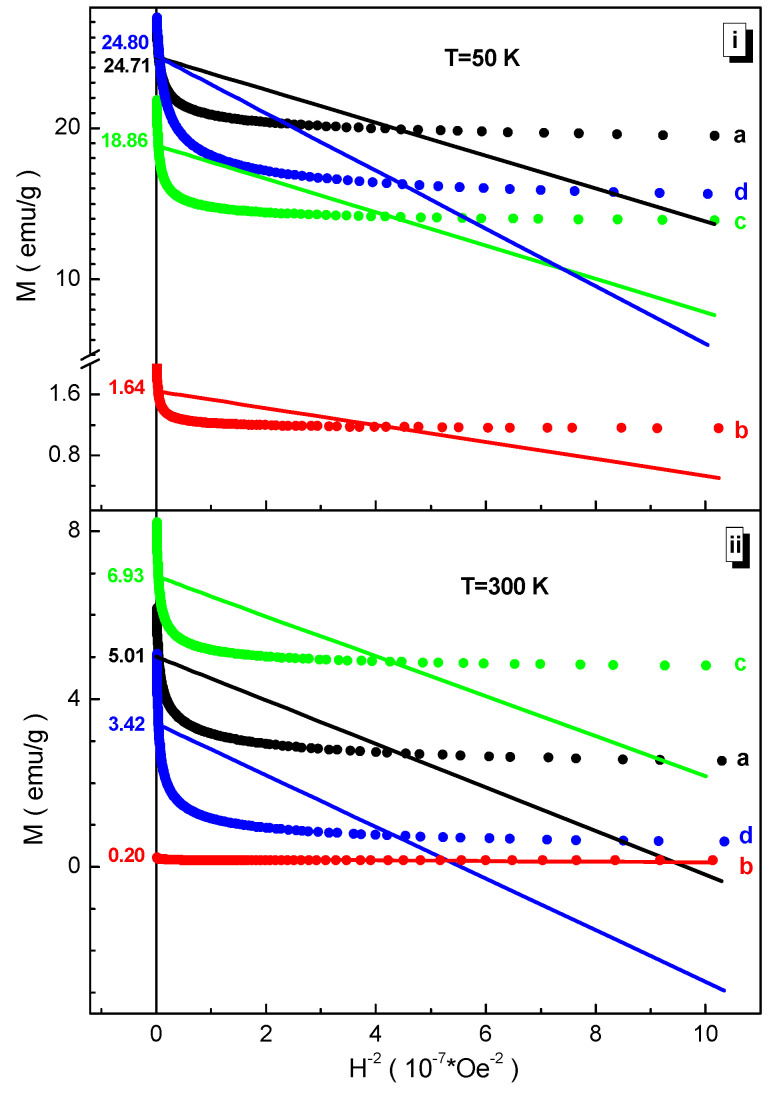
Dependencies of magnetization vs. inverse field square and their linear extrapolations at 50 K (**i**) and 300 K (**ii**) for the compositions (a) BaFe_5.84_Ga_6.16_O_19_, (b) BaFe_6.26_Al_2.27_Cr_3.47_O_19_, (c) BaFe_6.11_Al_1.56_Cr_2.17_Ga_2.16_O_19_, (d) BaFe_6.19_Al_1.25_Cr_1.57_Ga_1.74_In_1.26_O_19_.

The Law of Approach to Saturation [53] assumes that a linear extrapolation of the M(H^−2^) magnetization plotted against the inverse square of the magnetic field strength to the point of intersection with the *y*-axis will give the value of the M_s_ saturation magnetization. In this case, the field dependence of the magnetization should be chosen in the mode of decreasing the field from its maximum values. The definition region of the magnetic field is chosen depending on the curvature of the M(H) magnetization field dependence. The data analysis of the field dependencies of the magnetization at two temperatures using this rule are presented in Figure 6. It is clearly seen that the saturation magnetization at 50 K decreases from ~24.80 emu/g for the BaFe_6.19_Al_1.25_Cr_1.57_Ga_1.74_In_1.26_O_19_ (d) composition down to ~1.64 emu/g for the BaFe_6.26_Al_2.27_Cr_3.47_O_19_ (b) composition. At 300 K, the nature of the change in the M_s_ saturation magnetization is somewhat different. It decreases from ~6.93 emu/g for the BaFe_6.11_Al_1.56_Cr_2.17_Ga_2.16_O_19_ (c) composition down to ~0.20 emu/g for the BaFe_6.26_Al_2.27_Cr_3.47_O_19_ (b) composition.

When analyzing the data in Figure 6, three features can immediately be distinguished. The first feature is that although all four compositions contain 50% Fe^3+^ iron cations in the initial positions, the M_s_ saturation magnetization is different for all of them. This is mainly explained by the different 〈Fe^3+^(Al^3+^, Cr^3+^, Ga^3+^, In^3+^)–O^2−^〉 average bond lengths of the cations participating in the indirect superexchange interaction. The 〈Fe^3+^(Al^3+^, Cr^3+^, Ga^3+^, In^3+^)–O^2−^〉 average bond length of the indirect superexchange interaction, in the zeroth approximation, is proportional to the unit cell volume, the data for which are presented in Figure 2.

The second feature is the very low value of the M_s_ saturation magnetization for all the studied compositions compared to the BaFe_12_O_19_ initial compound. According to Gorter’s model [54], the maximum magnetic moment per formula unit in the ground state for this compound is 20 μ_B_/f.u., since there is a specific ordering of spins in non-equivalent crystallographic positions. Since the spins in positions 2a, 2b, and 12k are opposite to the spins in positions 4f_4_ and 4f_6_, then the total saturation moment, taking into account the degeneracy multiplicity of each position, is 20 μ_B_/f.u., which corresponds to ~100 emu/g for the initial unsubstituted BaFe_12_O_19_ barium hexaferrite. The low values of the M_s_ saturation magnetization of the studied compositions are explained mainly by the diamagnetic dilution of the iron sub-lattice, although the 〈Fe^3+^(Al^3+^, Cr^3+^, Ga^3+^, In^3+^)–O^2−^〉 average bond length of the indirect superexchange interaction is shorter than that of the initial BaFe_12_O_19_ barium hexaferrite. The action of the effect of diamagnetic dilution is stronger than the effect of the convergence of exchange centers.

The third feature is that the lowest value of the M_s_ saturation magnetization has the composition BaFe_6.26_Al_2.27_Cr_3.47_O_19_ (b) with the lowest value of the 〈Fe^3+^(Al^3+^, Cr^3+^, Ga^3+^, In^3+^)–O^2−^〉 average bond length, as can be seen from Figure 2. This is due to the number and type of substituting diamagnetic cations. A large number equal to three substituting cations with an ionic radius less than the ionic radius of the Fe^3+^ iron cation leads not only to the convergence of exchange centers, which should lead to an increase in the exchange interaction, but also to a significant distortion of the unit cell, and hence to a significant decrease in the 〈Fe^3+^(Al^3+^, Cr^3+^, Ga^3+^, In^3+^)–O^2−^–Fe^3+^(Al^3+^, Cr^3+^, Ga^3+^, In^3+^)〉 average bond angle from 180 degrees, which leads to a weakening of the exchange interaction. The effect of decreasing the 〈Fe^3+^(Al^3+^, Cr^3+^, Ga^3+^, In^3+^)–O^2−^–Fe^3+^(Al^3+^, Cr^3+^, Ga^3+^, In^3+^)〉 average bond angle dominates the effect of decreasing the 〈Fe^3+^(Al^3+^, Cr^3+^, Ga^3+^, In^3+^)–O^2−^〉 average bond length. Additionally, of course, a decrease in the values of the M_s_ saturation magnetization with increasing temperature seems to be natural, which is explained by the increasing disordering effect of thermal energy.

**Figure 7:**
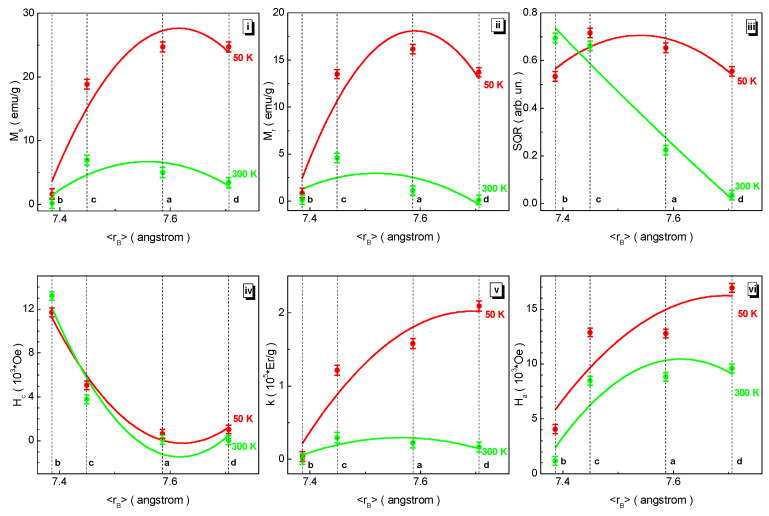
Dependencies of main magnetic parameters such as the M_s_ saturation magnetization (**i**), M_r_ residual magnetization (**ii**), SQR hysteresis loop squareness (**iii**), H_c_ coercivity (**iv**), k magnetic crystallographic anisotropy coefficient (**v**), and H_a_ anisotropy field (**vi**) at 50 K (red symbols) and 300 K (green symbols) vs. 〈r_B_〉 B sub-lattice average radius for the compositions (a) BaFe_5.84_Ga_6.16_O_19_, (b) BaFe_6.26_Al_2.27_Cr_3.47_O_19_, (c) BaFe_6.11_Al_1.56_Cr_2.17_Ga_2.16_O_19_, (d) BaFe_6.19_Al_1.25_Cr_1.57_Ga_1.74_In_1.26_O_19_.

Almost all the dependencies in Figure 7 are non-monotonic with an extremum point or a trend towards it, except for the dependence of the SQR loop squareness at 300 K. The M_s_ saturation magnetization curves for both temperatures are curved upwards. The maximum points of ~24.71 emu/g at 50 K and ~5.01 emu/g at 300 K in the M_s_ saturation magnetization are observed for the BaFe_5.84_Ga_6.16_O_19_ (a) composition. The relative change in the M_s_ saturation magnetization is ~94% for both temperatures. The M_r_ residual magnetization curves for both temperatures are also curved upwards. The maximum points of the dependencies of the M_r_ residual magnetization of ~16.16 emu/g at 50 K and ~1.12 emu/g at 300 K are also observed for the BaFe_5.84_Ga_6.16_O_19_ (a) composition. The relative change in the M_r_ residual magnetization under these conditions is ~94% at 50 K and −17% at 300 K, respectively. The dependencies of the SQR(〈r_B_〉) loop squareness have a heterogeneous character at different temperatures. At 50 K, this dependence is non-monotonic and has a maximum point of 0.65 arb.un. for the BaFe_5.84_Ga_6.16_O_19_ (a) composition. At 300 K, this dependence is monotonically decreasing and almost linear. The relative change in the SQR loop squareness is ~4% at 50 K and −189% at 300 K. The H_c_ coercivity curves for both temperatures are curved downwards. The minimum points of ~0.64 kOe at 50 K and ~0.10 kOe at 300 K in the M_s_ saturation magnetization are observed for the BaFe_5.84_Ga_6.16_O_19_ (a) composition. The H_c_ coercivity at 50 K relatively changes by −104%, while at 300 K it changes by −2390%.

In the case of uniaxial anisotropy, the k magnetic crystallographic anisotropy coefficient is proportional to the M_s_ saturation magnetization and the square root of the tangent of the slope of the linear extrapolation of the magnetization plotted as a function of the reciprocal square of the field k = 1.94* × M_s_ × [tan(φ)]^0.5^ [55]. This physical quantity was computed for both temperatures and plotted in Figure 7. These curves for both temperatures are curved upwards. At 50 K, the k magnetic crystallographic anisotropy coefficient exhibits a maximum point of ~2.09 × 10^5^ Erg/g for the BaFe_6.19_Al_1.25_Cr_1.57_Ga_1.74_In_1.26_O_19_ (d) composition and relatively changes by ~98%. At 300 K, it has a maximum point of ~0.22 × 10^5^ Erg/g for the BaFe_5.84_Ga_6.16_O_19_ (a) composition and changes by ~99%.

The H_a_ anisotropy field is directly proportional to the k magnetic crystallographic anisotropy coefficient and inversely proportional to the M_s_ saturation magnetization H_a_ = 2*k/M_s_ [56]. This physical quantity was also computed for both temperatures and plotted in Figure 7. These curves for both temperatures are curved upwards. The maximum points of ~16.92 kOe and ~9.61 kOe are fixed at 50 K and 300 K, respectively, for the BaFe_6.19_Al_1.25_Cr_1.57_Ga_1.74_In_1.26_O_19_ (d) composition. The H_a_ anisotropy field has the relative change of ~76% and ~88% at 50 K and 300 K, respectively.

**Figure 8:**
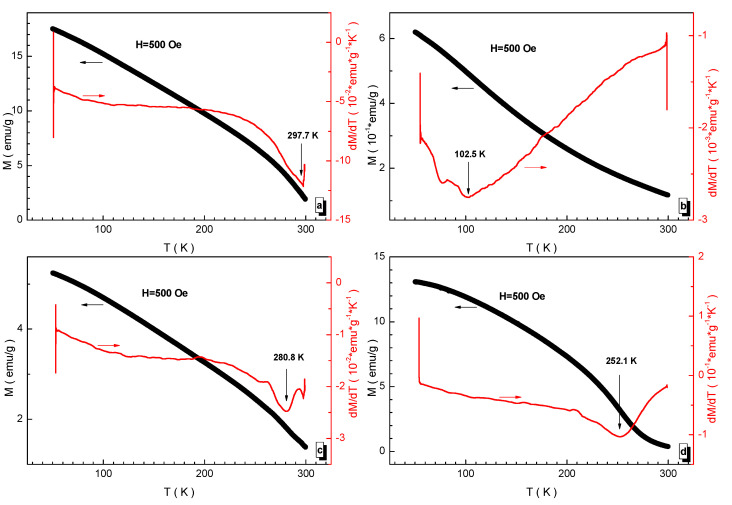
Temperature dependencies of magnetization (black symbols and left axis) in field of 500 Oe and its derivative (red symbols and right axis) for the compositions (**a**) BaFe_5.84_Ga_6.16_O_19_, (**b**) BaFe_6.26_Al_2.27_Cr_3.47_O_19_, (**c**) BaFe_6.11_Al_1.56_Cr_2.17_Ga_2.16_O_19_, (**d**) BaFe_6.19_Al_1.25_Cr_1.57_Ga_1.74_In_1.26_O_19_.

The temperature behavior of the magnetization is measured in a field of 500 Oe and it is shown in Figure 8. All the compositions experience an almost complete transition to the paramagnetic state up to 300 K. It should be noted that the transition temperature range is rather wide, which is typical for systems that are not completely homogeneous or frustrated. The transition temperature was determined from the point of minimum of the first derivative of magnetization with respect to temperature. It should also be noted that even the highest T_mo_ magnetic ordering temperature of ~297.7 K recorded for the BaFe_5.84_Ga_6.16_O_19_ (a) composition is significantly lower, by more than two times, than the Curie temperature of ~730 K [13] for the initial BaFe_12_O_19_ barium hexaferrite.

**Figure 9:**
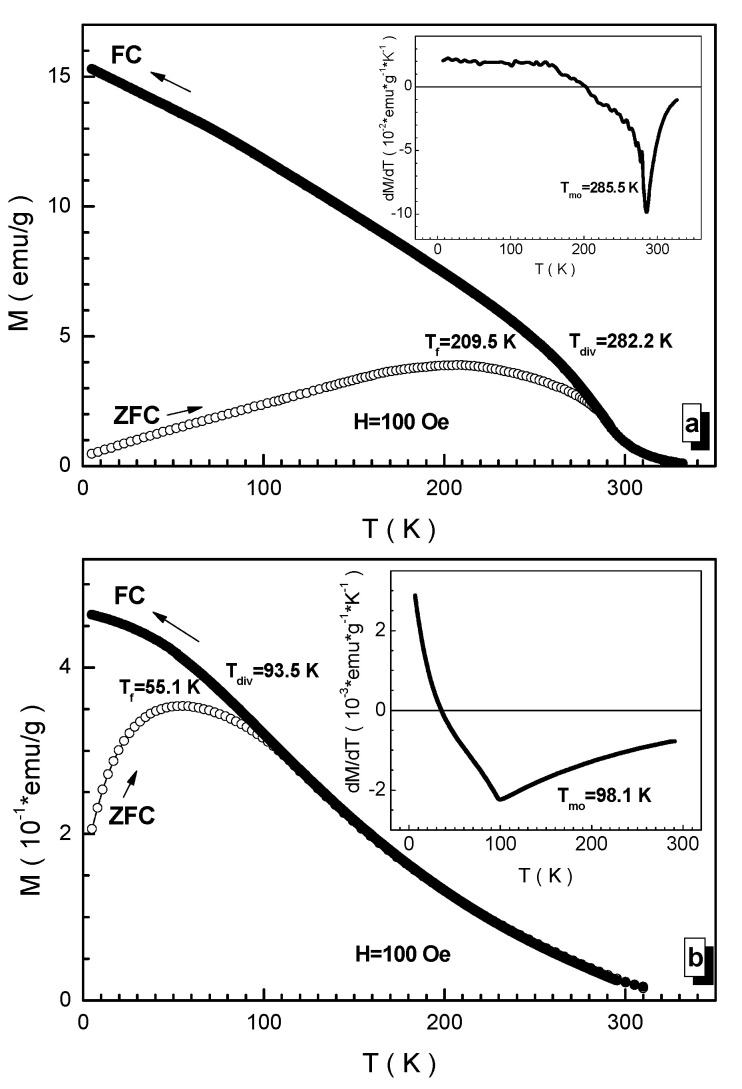
Temperature dependencies of magnetization in ZFC (open symbols) and FC (full symbols) regimes in field of 100 Oe for the compositions (**a**) BaFe_5.84_Ga_6.16_O_19_, (**b**) BaFe_6.26_Al_2.27_Cr_3.47_O_19_. Insets demonstrate the temperature derivatives of ZFC curves. The critical temperatures are defined.

Experimentation into the understanding of the nature of magnetic interactions of multisubstituted barium hexaferrites was carried out in the process of measuring the magnetization in a weak magnetic field in the so-called ZFC and FC modes. Measurements in such regimes make it possible to establish the spin dynamics and the magnetic state of the compositions under study. From Figure 9, a set of critical temperatures can be determined. Figure 9 clearly shows that the ZFC and FC curves differ significantly below the T_div_ divergence temperature. The FC curve tends to saturation, while the ZFC curve experiences a drop after reaching a maximum. The temperature of the maximum of the ZFC curve is the T_f_ freezing temperature of the spins. It is ~209.5 K for the BaFe_5.84_Ga_6.16_O_19_ (a) composition and ~55.1 K for the BaFe_6.26_Al_2.27_Cr_3.47_O_19_ (b) composition. This temperature determines, according to the Bean–Livingston model [57], the average diameter of a magnetically ordered cluster in a magnetically disordered matrix. According to this, model representations of a region with a frustrated orientation of spins are located in a medium with a long-range magnetic order of moments. According to the Bean–Livengston relation, the average size of such clusters varies up to ~200 nm [58]. At the same time, the T_div_ divergence temperature of the ZFC and FC curves determines the diameter of the largest magnetically ordered cluster and for these samples they are ~282.2 K and ~93.5 K (see Figure 9). The diameter of the maximum cluster reaches ~300 nm. The T_mo_ magnetic ordering temperatures in a field of 100 Oe have slightly lower values.

**Figure 10:**
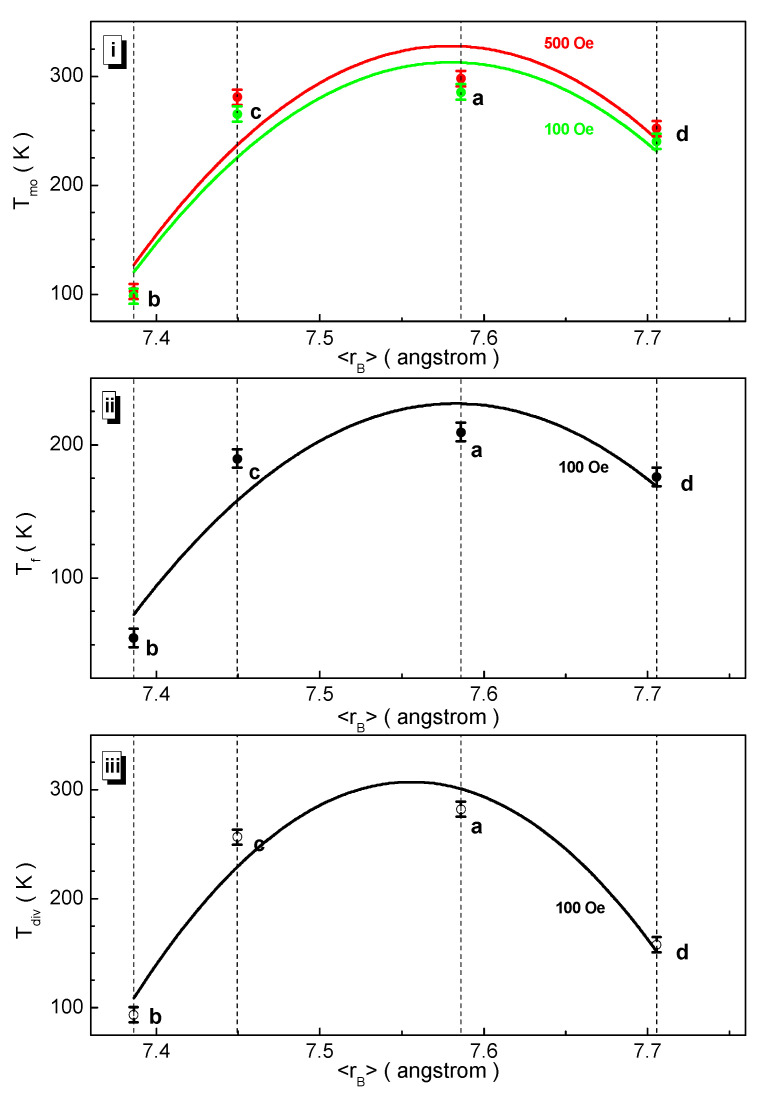
Dependencies of some critical magnetic temperatures such as the T_mo_ magnetic ordering temperature (**i**) defined in 100 Oe (green symbols) and 500 Oe (red symbols), T_f_ freezing temperature (**ii**), and T_div_ divergence temperature (**iii**) vs. 〈r_B_〉 B sub-lattice average radius for the compositions (a) BaFe_5.84_Ga_6.16_O_19_, (b) BaFe_6.26_Al_2.27_Cr_3.47_O_19_, (c) BaFe_6.11_Al_1.56_Cr_2.17_Ga_2.16_O_19_, (d) BaFe_6.19_Al_1.25_Cr_1.57_Ga_1.74_In_1.26_O_19_.

The dependencies of the critical temperatures on the B-sub-lattice average ionic radius presented in Figure 10 are non-monotonic and curved upwards. The maximum points of T_mo_(100 Oe) = 285.5 K, T_mo_(500 Oe) = 297.7 K, T_f_ = 209.5 K, and T_div_ = 282.2 K are observed for the BaFe_5.84_Ga_6.16_O_19_ (a) composition. The T_mo_ magnetic ordering temperature relatively changes by 59% in 100 Oe and 500 Oe. The T_f_ freezing temperature relatively changes by 69%. The T_div_ divergence temperature experiences a relative change of 41%.

Thus, the multisubstitution of the Fe^3+^ iron cations mainly by the Al^3+^, Cr^3+^, Ga^3+^, and In^3+^ diamagnetic cations leads to a weakening of the Fe^3+^(Al^3+^, Cr^3+^, Ga^3+^, In^3+^)–O^2−^–Fe^3+^(Al^3+^, Cr^3+^, Ga^3+^, In^3+^) indirect superexchange interactions and the formation of a frustrated magnetic state, which is an inhomogeneous arrangement of magnetically disordered nano-sized clusters in a magnetically ordered matrix. Although the radius of the substituting cations is less than the radius of the iron cation, which leads to a decrease in the 〈Fe^3+^(Al^3+^, Cr^3+^, Ga^3+^, In^3+^)–O^2−^〉 average bond length, this does not lead to an increase in the intensity of indirect superexchange interactions. This is due to the inhomogeneous distribution of substituting cations, the diamagnetic nature of these cations, as well as a strong distortion of the unit cell.

## 4. Conclusions

A series of the barium hexaferrite solid solutions with a magnetoplumbite structure was obtained by solid-phase synthesis, in which some of the iron atoms (about 50%) are substituted by three-charged Al^3+^, Cr^3+^, Ga^3+^, and In^3+^ cations of different elements, while the number of substituting cations increases from one to four, and their concentrations in the total number are close. The results of the work on the synthesis give grounds to believe that the use of a complex of dopants compared to substitution with one cation makes it possible to achieve deeper degrees of iron substitution. From the point of view of the thermodynamics of phase equilibria, the greater stability of phases with multicomponent substitution can be explained by an increase in the configurational mixing entropy of the sub-lattice formed by the Fe^3+^ iron cations, which leads to a decrease in the Gibbs energy of the phase as a whole. From the crystallography point of view, the presence of a large number of substituents with ionic radii both larger (In^3+^) than that of the Fe^3+^ iron cation and smaller (Al^3+^, Cr^3+^, and Ga^3+^) ones leads to the fact that the unit cell parameters of the multicomponent structure, on average, approach the parameters of the initial BaFe_12_O_19_ barium hexaferrite. The values of the configurational mixing entropy were calculated for all the compositions. A slight deviation of the chemical composition of the obtained solid solutions from the nominal value was established by the energy-dispersive X-ray spectroscopy method. The phase purity and values of the unit cell parameters were refined from X-ray scattering data using full-profile analysis in the Rietveld method. A non-monotonic behavior of the unit cell parameters as a function of the B-sub-lattice average ionic radius of the magnetoplumbite structure was found. A minimum unit cell volume of ~667.15 Å^3^ was found for the composition BaFe_6.11_Al_1.56_Cr_2.17_Ga_2.16_O_19_ with a B-sub-lattice average ionic radius of ~7.449 Å. The average crystallite size varied within 5.5–6.5 μm. The temperature and field dependencies of the magnetization have been measured. The values of the saturation magnetization, residual magnetization, hysteresis loop squareness, and coercivity at 50 K and 300 K were extracted from the experimental data. Using the Law of Approach to Saturation, the magnetic crystallographic anisotropy coefficient and anisotropy field were calculated. Multisubstitution leads to a significant decrease in such magnetic parameters as the Curie temperature and spontaneous magnetization at both temperatures. A maximum Curie temperature of ~297.7 K was found for the composition BaFe_5.84_Ga_6.19_O_19_ with a B-sub-lattice average ionic radius of ~7.586 Å in a field of 500 Oe. A maximum saturation magnetization of ~24.7 emu/g was found for the composition BaFe_5.84_Ga_6.19_O_19_ with a B-sub-lattice average ionic radius of ~7.586 Å at 50 K. A maximum hysteresis loop squareness of ~0.72 was found for the composition BaFe_6.11_Al_1.56_Cr_2.17_Ga_2.16_O_19_ with an average ionic radius of ~7.449 Å at 50 K. A maximum magnetic crystallographic anisotropy coefficient of ~2.09 × 10^5^ Erg/g was found for the composition BaFe_6.19_Al_1.25_Cr_1.57_Ga_1.74_In_1.26_O_19_ with a B-sub-lattice average ionic radius of ~7.706 Å at 50 K. The frustrated magnetic state including the nano-sized clusters with an average diameter in the range of 50–200 nm was established from the results of measuring the ZFC and FC temperature magnetizations. The multisubstitution of the Fe^3+^ iron cations mainly by the diamagnetic cations leads to a weakening of the Fe^3+^(Al^3+^, Cr^3+^, Ga^3+^, In^3+^)–O^2−^–Fe^3+^(Al^3+^, Cr^3+^, Ga^3+^, In^3+^) indirect superexchange interactions and the formation of a frustrated magnetic state, which is an inhomogeneous arrangement of magnetically disordered nano-sized clusters in a magnetically ordered matrix. This type of substitution leads to a decrease in the 〈Fe^3+^(Al^3+^, Cr^3+^, Ga^3+^, In^3+^)–O^2−^〉 average bond length, however, this does not lead to an increase in the intensity of indirect superexchange interactions. This is due to the inhomogeneous distribution of substituting cations, the diamagnetic nature of these cations, as well as a strong distortion of the unit cell. The interpretation of the obtained experimental data is carried out taking into account the increased stability of high-entropy phases and regular changes in the intensity of the Fe^3+^(Al^3+^, Cr^3+^, Ga^3+^, In^3+^)–O^2−^–Fe^3+^(Al^3+^, Cr^3+^, Ga^3+^, In^3+^) indirect superexchange interactions as a result of magnetic dilution of the iron sub-lattice in the magnetoplumbite structure.

## Figures and Tables

**Table 1:** Compositions (wt.%) of the initial mixture.

No	Chemical Formula	Mass Percentage of the Original Component, %						Smix
1.	BaFe_6_Al_6_O_19_	BaCO_3_	Fe_2_O_3_			Al_2_O_3_		0.693147
		20.09	48.77			31.14		
2.	BaFe_6_Cr_6_O_19_	BaCO_3_	Fe_2_O_3_			Cr_2_O_3_		0.693147
		17.43	42.31			40.27		
3.	BaFe_6_Ga_6_O_19_	BaCO_3_	Fe_2_O_3_			Ga_2_O_3_		0.693147
		15.93	38.67			45.39		
4.	BaFe_6_Al_3_Cr_3_O_19_	BaCO_3_	Fe_2_O_3_	Al_2_O_3_		Cr_2_O_3_		1.039721
		18.66	45.31	14.46		21.56		
5.	BaFe_6_Al_2_Gr_2_Ga_2_O_19_	BaCO_3_	Fe_2_O_3_	Al_2_O_3_	Cr_2_O_3_	Ga_2_O_3_		1.242453
		17.66	42.86	9.12	13.60	1.04		
6.	BaFe_6_Al_1.5_Cr_1.5_Ga_1.5_In_1.5_O_19_	BaCO_3_	Fe_2_O_3_	Al_2_O_3_	Cr_2_O_3_	In_2_O_3_	Ga_2_O_3_	1.386294
		16.40	39.80	6.35	9.47	16.30	11.68	

**Table 2:** Unit cell parameters of the obtained solid solutions.

No	Chemical Formula and Label	Unit Cell Parameters		
		*a*, Å	*c*, Å	V, Å^3^
1.	BaFe_5.84_Ga_6.19_O_19_ (a)	5.8515(2)	23.1460(19)	686.33(7)
2.	BaFe_6.26_Al_2.27_Cr_3.47_O_19_ (b)	5.8077(14)	22.8396(7)	667.15(2)
3.	BaFe_6.11_Al_1.56_Cr_2.17_Ga_2.16_O_19_ (c)	5.8148(13)	22.9660(5)	672.47(19)
4.	BaFe_6.19_Al_1.25_Cr_1.57_Ga_1.74_In_1.26_O_19_ (d)	5.8884(2)	23.3191(12)	700.23(5)
5.	BaFe_12_O_19_ [3]	5.8930(3)	23.1940(2)	697.50(13)

**Table 3:** Elemental composition of the obtained samples.

Elemental Composition of the Obtained Samples, Atom %						Gross Formula and Label
BaFe_6_Ga_6_O_19_						BaFe_5.84_Ga_6.16_O_19_ (a)
Ba		Fe	Ga			
3.3		20.1	21.3			
BaFe_6_Al_3_Cr_3_O_19_						BaFe_6.26_Al_2.27_Cr_3.47_O_19_ (b)
Ba		Fe	Al	Cr		
4.0		21.4	7.8	11.9		
BaFe_6_Al_2_Cr_2_Ga_2_O_19_						BaFe_6.11_Al_1.56_Cr_2.17_Ga_2.16_O_19_ (c)
Ba		Fe	Al	Cr	Ga	
3.7		21.0	5.4	7.5	7.4	
BaFe_6_Al_1.5_Cr_1.5_Ga_1.5_In_1.5_O_19_						BaFe_6.19_Al_1.25_Cr_1.57_Ga_1.74_In_1.26_O_19_ (d)
Ba	Fe	Al	Cr	Ga	In	
3.5	21.4	4.3	5.4	6.0	4.4	

## Data Availability

Not applicable.
